# Embodied strategies for public speaking anxiety: evaluation of the Corp-Oral program

**DOI:** 10.3389/fnhum.2023.1268798

**Published:** 2023-11-27

**Authors:** Alfonso García-Monge, Santiago Guijarro-Romero, Eduardo Santamaría-Vázquez, Lucio Martínez-Álvarez, Nicolás Bores-Calle

**Affiliations:** ^1^Department of Didactic of Musical, Plastic and Corporal Expression, University of Valladolid, Valladolid, Spain; ^2^Embodied Education Research Group, University of Valladolid, Valladolid, Spain; ^3^Biomedical Engineering Group, University of Valladolid, ETSIT, Valladolid, Spain; ^4^Centro de Investigación Biomédica en Red de Bioingeniería, Biomateriales y Nanomedicina (CIBER-BBN), Valladolid, Spain

**Keywords:** public speak anxiety, embodied pedagogy, embodied strategies, body awareness, teacher training, teachers’ embodied experience, EEG

## Abstract

**Background:**

Public speaking is an indispensable skill that can profoundly influence success in both professional and personal spheres. Regrettably, managing anxiety during a speech poses a significant challenge for many of the population. This research assessed the impacts of a Corp-Oral program, designed to manage public speaking anxiety in university students, based on, body awareness, embodied message techniques, simulation, embodied visualization, body transformation, and gesture enhancement.

**Methods:**

Thirty-six students (61% women; M_*age*_ = 20.22, SD = 1.23 years) were randomly assigned to either an experimental group (*n* = 18), which underwent the Corp-Oral program, or a control group (*n* = 18). Self-perceived anxiety, heart rate, and electroencephalography were measured in a pre-test and a post-test.

**Results:**

The study reveals that the Corp-Oral program significantly (*p* < 0.005) reduced both physiological responses (heart rate) and self-reported measures of anxiety. The alteration was more noticeable in self-reported anxiety measures (a decrease of 33.217%) than in heart rate (a decrease of 4.659%). During the speech, the experimental group exhibited increased cortical activation in areas related to emotional regulation, consciousness, sensorimotor integration, and movement control. A significant increase in frontal alpha asymmetry was observed for the experimental group in the post-test, but there were no significant variations in the theta/beta ratio.

**Conclusion:**

These findings underline the benefit of managing public speaking anxiety not merely by reducing it but by channeling it through embodied strategies. These strategies could lead to greater action awareness that would cushion the physiological effect of the anxiety response and help generate a better self-perception of the anxiety state.

## 1 Introduction

The objective of this brief research report is to present some findings from the evaluation of a program called Corp-Oral, which focuses on managing public speaking anxiety, guided by the question: What are the effects in self-perceived anxiety, heart rate, and electroencephalographic registers of a program based on body awareness, embodied message techniques, simulation, embodied visualization, body transformation, and gesture enhancement?

Public speaking skills are crucial for academic and professional success (Campbell, 2009). Individuals with strong communication skills are more likely to graduate from university and attain leadership positions than their counterparts (Schneier, 2006). Many businesses rank communication skills among the most important when hiring staff (Marzo Navarro et al., 2006; Hernández-March et al., 2009; Tan and Laswad, 2018; Coffelt et al., 2019; Hill et al., 2019). However, public speaking anxiety (PSA) is one of the most common social fears faced by the population (Stein et al., 1994), and it directly impacts their academic and professional success (Ruscio et al., 2008).

Many teachers (especially those new to the profession) and students suffer from PSA to varying degrees when facing public speaking situations (Orejudo et al., 2005; Maldonado and Reich, 2013; Morales Rodríguez et al., 2014; García Fernández et al., 2015; Raja, 2017). At best, it can distort the message; at worst, it can be incapacitating (Overholser, 2002). The physiological reaction to stress triggers the hypothalamic-pituitary-adrenocortical (HPA) axis, increasing the modulation of the sympathetic autonomic system. This prepares the body for an impending stressful event, such as a public speaking engagement, causing a condition known as anticipatory anxiety (Beltrán-Velasco et al., 2019). Furthermore, this reaction releases chemical messengers that influence various brain areas responsible for memory and learning. This chain of events can interfere with normal neuronal operations in the prefrontal cortex, leading to disruptions in critical cognitive functions, including memory retention, decision-making processes, and learning ability (Hood et al., 2015). An increased situational awareness (e.g., Grossman et al., 2004; Vago and Silbersweig, 2012) or a transformation of the threat into a challenge (Blascovich et al., 1999), could modulate these reactions of the sympathetic autonomic system.

These physiological changes have led to the use of various devices to detect public speaking anxiety by recording changes in heart rate (Croft et al., 2004), blood oxygen saturation (Mocny-Pachońska et al., 2021), skin conductance (Kimani and Bickmore, 2019), or cortical activity (Miskovic et al., 2010).

A consequence of the activation produced in situations of anxiety is an increase in heart rate (HR); thus, numerous studies have used it as a reliable indicator to assess anxiety (Behnke et al., 1974; Croft et al., 2004; Zeidan et al., 2010; Stevens et al., 2011; von Dawans et al., 2011). Similarly, increased sweating in such situations leads to increased skin conductance (Barry and Sokolov, 1993; Eckman and Shean, 1997). Regarding cortical activity measured with electroencephalography (EEG), public speaking anxiety is related to an increase in high beta spectral power (20–30 Hz) (Spitzer and Haegens, 2017; Díaz et al., 2019) cross-frequency spectral coupling between slow and fast wave activity in the right frontal positions, an increase in the gamma band power during the speech anticipation condition, in the parietal electrode sites (Miskovic et al., 2010); a decrease in the frontal theta/beta ratio (Poole et al., 2021, since higher levels of the theta/beta ratio may be indicative of reduced cognitive control, van Son et al., 2019); and increased frontal alpha asymmetry (Hofmann et al., 2005; Yang et al., 2013; Wang et al., 2015).

### 1.1 Corp-Oral program

There are many techniques and therapies to address public speaking anxiety issues: based on cognitive-behavioral treatments with exposure to real presentation situations (Overholser, 2002; Ponniah and Hollon, 2008; Finn et al., 2009) or in virtual environments (van Minnen and Foa, 2006; Takac et al., 2019; Reeves et al., 2022; Lim et al., 2023); focused on breathing techniques (Dincer et al., 2022) and mindfulness (Galantino et al., 2005; Zeidan et al., 2010); or those that aim to generate new dispositions through visualizations of future positive situations (McEvoy et al., 2015; Landkroon et al., 2022) or rewriting adverse memories of past situations (Morina et al., 2017).

The Corp-Oral program borrows some of the techniques mentioned above but reinterprets them, emphasizing the participants’ embodied experience. The program focuses on the following aspects: improving body awareness and control, enhancing body language registers (mastering contrasts, movement qualities, and different body attitudes), embodying messages, improving gestures to support oral discourse, identifying bodily sensations in a state of anxiety, and transforming that discomfort into communicative energy.

The program is based on the following ideas: The way each individual interprets anxiety can determine whether it serves as a mechanism to enhance alertness and activity or, alternatively, act as a constraint to their performance (Baumeister et al., 2013; Crum et al., 2013; Brooks, 2014). In the Corp-Oral program, participants are encouraged to physically re-label the sensations of public speaking anxiety (turning threat into a challenge, Blascovich et al., 1999) and redirect that energy to emphasize messages, increase expressiveness, and experience exposure situations as an opportunity to share their work.

Changes in gesture and the redirection of emotions have been widely studied (Price et al., 2012; Carney et al., 2015; Nair et al., 2015; Harvey et al., 2020; Körner and Schütz, 2020; Awad et al., 2021). This program works on changing expressive registers with the body and generating new gestures supported by new emotional states to face public speaking.

The effect of different body techniques on modulating anxiety and stress has been studied (Bräuninger, 2012; Felsman et al., 2019; Kronsted, 2020; Peper et al., 2022). Many studies have shown the role of self-awareness in modulating the effects produced by the sympathetic autonomic system (e.g., Grossman et al., 2004; Vago and Silbersweig, 2012). The Corp-Oral program uses body and expressive techniques, such as pantomime or dramatization, to expand the participants’ expressive repertoire and embody verbal messages so that gestures gain awareness, intentionality, energy, and coherence with oral discourse.

Furthermore, imaginary practice has also proven suitable for addressing anxiety problems (Ayres and Hopf, 1992; Pile et al., 2021). In the Corp-Oral program, embodied visualizations are performed in which an attempt is made to change the participant’s gestures, generating feelings of energy, optimism, and gratitude (Fredrickson, 2000), and new bodily sensations that transform discomfort into competence and communicative emphasis.

In summary, this program aims to help participants experience themselves in a more positive way in public speaking situations through various simulations, body awareness, embodied visualization, body transformation, and gesture enrichment tasks. In this brief research report, we present some findings about the effects of this program in self-perceived anxiety, heart rate and EEG activity.

## 2 Materials and methods

### 2.1 Participants

The sample size was calculated, assuming a medium effect size (given the exploratory nature of our study and the absence of this data in previous research on this topic), a significance level of 0.05, and a power of 0.80 (Serdar et al., 2021). The calculation suggested that a total sample size of 36 participants (18 per group) would be adequate to detect an effect of this magnitude with appropriate statistical power. In total, 36 students aged between 19 and 23 (M_*age*_ = 20.22, SD = 1.23 years) participated. Of these, 18 participated in the Corp-Oral program (12 women, 6 men), and the remaining 18 formed the control group (10 women, 8 men).

The students were enrolled in the 2nd and 4th year of the Teacher Training degree program. Selection criteria included students who admitted to experiencing public speaking anxiety and scored above 98 (indicative of moderate to high anxiety) on the Personal Report of Public Speaking Anxiety (PRPSA) (McCroskey, 1970).

The selected students were randomly assigned to either the control or experimental group. The control group (in small groups of three to six participants) received a 40-min lecture about the neurophysiology of anxiety, the function of anxiety, and the potential to harness it to enhance the response to situations such as public speaking. Following that, for 20–30 min, participants were informed about the role of breathing in addressing anxiety, and a few breathing exercises were conducted (Howe and Dwyer, 2007). The experimental group participated in the Corp-Oral program. The program spanned five continuous hours, all conducted within a single day and was conducted in small groups (ranging from three to six individuals) in a face-to-face setting, while trying to adapt to the individual needs of the participants, following the subsequent scheme of activities: a) Introduction to the workshop and its content; b) overview of the neurophysiology of anxiety, its function, and the potential to harness it for our benefit; c) exercises for presentation and body disinhibition; d) body control exercises, contrasting different parameters of movement qualities, body attitudes, and shifts in communicative expression; e) exercises to embody verbal messages and enhance communicative emphasis using different body parts; f) visualization exercises for a public exposure situation, emphasizing bodily transformation and redirecting feelings of anxiety into energy to underscore messages, heighten expressivity, and approach the situation as a positive challenge, fostering feelings of vitality, optimism, and gratitude; g) preparation, rehearsal, and presentation in front of the group of a brief text, followed by a group analysis of the presentation; h) exposure exercise facing disapproving or adversarial expressions from the audience, focusing on the bodily sensations generated during visualization, and group analysis, and i) final conclusions.

Three students from the control group did not participate in the post-test and were consequently excluded from the analyses (Supplementary Figure 1). Participants provided informed consent and retained the right to withdraw from the study without penalty. The Research Ethics Committee of the University of Valladolid approved the study.

### 2.2 Measures

Different procedures (self-report, behavioral, and physiological measures) were used to gather information during the pre and post-tests.

#### 2.2.1 Self-report

The PRPSA, validated by McCroskey (1970) and Mörtberg et al. (2018), was used for the initial selection of participants. It consists of a 34-item scale (respondents rate each item on a 5-point scale: Strongly Disagree = 1; Disagree = 2; Neutral = 3; Agree = 4; Strongly Agree = 5) where three scoring intervals determine the level of public speaking anxiety (High, between 131 and 170; Low, from 34 to 98; and Moderate, between 98 and 131). Compared to other scales, PRPSA more directly addresses the fear of public speaking in educational settings.

The Subjective Unit of Distress (SUDS) was used to gauge subjective anxiety at different points during the pre-test and post-test (Wolpe, 1990; Tanner, 2012). Through a single question (How anxious do you feel?), the intensity of self-perceived anxiety (SPA) was measured, ranging from 0 (no anxiety) to 10 (extreme anxiety). Due to the simplicity of this procedure, it can be applied successively throughout the process and used to monitor anxiety feeling variance over time, which explains its wide use in these types of studies (Croft et al., 2004; van Minnen and Foa, 2006; Gallego et al., 2022; Landkroon et al., 2022).

#### 2.2.2 Behavioral measure

Evaluators, in front of whom the participants gave their presentations, utilized a scale of Observed Evaluation of Confidence-Disturbance in Speech, but their results will not be included in this brief report.

#### 2.2.3 Physiological measures

Various physiological measures were collected for each phase of the pre-test and post-test (initial “Baseline” phase, speech “Preparation” phase, “Anticipation” phase, “Speech” phase, and “Recovery” phase).

An Embrace Plus device (Empatica Inc.; FDA-validated) was used to collect electrodermal activity data (sampling frequency: 4 Hz; range; [0.01; 100] μS) and heart rate (sampling frequency: 64 Hz). Heart rate (HR) was also collected through a CheckMe O_2_ + pulse oximeter (Wellue, Viatom Technology; sampling rate: 150 Hz), as well as oxygen saturation (SR = 1 Hz) (validated by various studies, e.g., Santos et al., 2022). Blood pressure data (not used in this brief research report) were collected through the Omron M7 Intelli IT device (Omron).

EEG data were recorded for each phase of the pre-test and post-test from 32-channels with Ag/AgCl sensors (EasyCap) using an Emotiv Epoc Flex gel kit at a sampling frequency of 128 Hz. Proper contact was verified through the EmotivPro program, which displays the contact quality status and the EEG quality status. Data were preprocessed through EEGLab for Matlab (Delorme and Makeig, 2004). High-pass (0.5 Hz) and low-pass (45 Hz) IIR Butterworth filters were applied. Data were cleaned of artifacts with an initial visual inspection, after which an artifact subspace reconstruction (ASR) algorithm was applied to discard channels silenced for more than 5 s or with high-frequency noise of more than 4 standard deviations. Subsequently, data were re-referenced by computing the average reference (CAR). Finally, independent component analysis (ICA) was applied, and components were discarded where non-neuronal sources predominated (artifacts), using the EEGLab plugin, ICALabel.

### 2.3 Procedure

**FIGURE 1:**
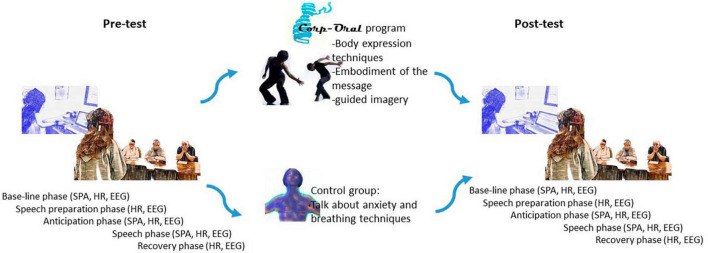
Procedure. SPA, self-perceived anxiety; HR, hear rate; EEG, electroencephalographic.

The structure of the “Simulated Public Speaking Test” (SPST) proposed by McNair et al. (1982) was followed for the conduct of the pre-test and post-test. Both for the post-test and the pre-test, two adjacent rooms were used. The baseline measurements were taken in one room, and the oral presentation test in front of a panel occurred in the other. The same protocol and phases were followed in both the pre-test and post-test (Figure 1).

Participants were received in the first room and briefed on the procedure and use of various devices. They were also requested to provide their informed consent after explaining the terms of their participation (they could withdraw from the process at any time, the confidentiality of the data, etc.). While the devices were being attached, participants completed self-report scales. Once good contact with the EEG signals was established, participants were shown their cortical activity and briefly explained the recorded signals and the effect of sudden movements and blinking on the distortion of the EEG record.

Following these explanations, an initial “Baseline” phase began where participants’ blood pressure was taken, and a 2-min record (EEG and HR) was made with eyes closed and 2 min with eyes open looking at a fixed point.

Upon completing this initial “Baseline” phase, the participant was told to prepare a 3-min speech about a lesson plan to be developed with schoolchildren (speech “Preparation” phase), which would be presented in front of a panel. Each participant was asked to prepare a 3-min speech in 3 min.

After the speech “Preparation” phase, there was a 3-min waiting period before moving to the room where the panel awaited (“Anticipation” phase). During these 3 min of waiting, blood pressure was retaken, and participants were again asked about their self-reported anxiety. During the post-test, the control group was reminded that they could perform the breathing exercises explained in a previous session. In contrast, the group that received the Corp-Oral program was invited to implement a technique of activation and redirection of anxiety by changing their bodily and emotional attitude (following what was worked on in the program).

After this “Anticipation” phase, participants were accompanied to the room with the panel for the “Speech” phase. The panel was composed of three teachers who were unfamiliar to the participants. Their expression was neutral, and they were not to make gestures of approval or disapproval. Participants stood 3 meters from the panel, in the center of the room (with no walls or furniture nearby). They were not allowed to have anything in their hands or use support papers.

Following the presentation, the participants were invited back to the first room, where immediately after “Speech phase” were asked about their SPA during the “Speech” phase. Additionally, new blood pressure measurements were taken. They were also asked about their bodily sensations during the speech (Figure 1).

### 2.4 Analysis

The normality of the SPA and heart rate (HR) data was evaluated using the Shapiro-Wilk test. The data did not show a normal distribution, and following Tan and Tabatabai (1985), mixed analyses of variance (ANOVA) were carried out to examine the effects of the 2 × 2 design with one between-groups factor (control vs. experimental) and one within-groups factor (time: pre-test vs. post-test). The magnitude of the effect was quantified using the generalized eta squared (*η*^2^*_*G*_*) measure, which is advocated for in the context of repeated-measures ANOVAs. According to Bakeman (2005), values of 0.02, 0.13, and 0.26 represent small, medium, and large effect sizes, respectively. The power analysis was calculated for the mixed ANOVA designs with repeated measures for two groups, with the number of measures ranging from one measure per participant in the case of SPA to 10 measures per participant for HR, assuming sphericity in the analysis.

The EEG data were segmented into 2-s epochs, and the absolute and relative spectral powers for the different frequency bands (delta 0.5–4 Hz, theta 4–8 Hz, alpha 8–13 Hz, low beta 13–20 Hz and high beta 20–30 Hz) were calculated for the various channels through fast Fourier transform (FFT). The EEGLab “Study” tool was used to carry out the comparisons between the pre-test and post-test. With it, the channels in which the most significant differences appeared were detected through permutations. The absolute and relative spectral powers were explored in these channels using Darbeliai plugin for EEGLab. The Shapiro-Wilk test showed a significant departure from normality, therefore, the Wilcoxon Sign-Ranked (WSR) test was applied to look for significant differences. Additionally, the frontal theta/beta ratios and the frontal alpha asymmetry (following the standard formula for FAA = ln(F4) - ln(F3), Allen et al., 2004) were calculated, and mixed analysis of variance was conducted on the results of these ratios.

This brief report only includes a few relevant results. The focus of SPA and HR is mainly on the “Baseline” (eyes open), “Anticipation,” and “Speech” phases, while the EEG data results concentrate on the differences during the “Speech” phase.

## 3 Results

The results of the mixed analyses of variance with the SPA measures indicated significant differences in the “Anticipation” and “Speech” phases (Table 1). Firstly, for the “Anticipation” phase, the interaction between Group and Test was significant (*F*(1,17) = 32.81, *p* < 0.001, *η*^2^_*G*_ = 0.295), implying that the training not only influenced anticipatory anxiety levels but also altered the trajectory of this anxiety over time. In the *post hoc* analysis, the experimental group (M*_*anticip pre*_* = 8.5, SD = 1.2; *M_*anticip post*_* = 5.79, SD = 0.8) showed a significant difference in SPA compared to the control group (M*_*anticip pre*_* = 8.57, SD = 1; *M_*anticip post*_* = 8.65, SD = 1.2) in the post-test (*F*(1,34) = 59.63, *p* < 0.001, *η*^2^_*G*_ = 0.637) with the experimental group, indicating diminished anxiety levels during anticipation after undergoing the training. Likewise, in “Speech” phase, the interaction between Group and Test was significant (*F*(1,17) = 63.47, *p* < 0.001, *η*^2^_*G*_ = 0.397), suggesting that the training effectively modulated the change in SPA over time. In the *post hoc* analysis, the experimental group (M*_*speech pre*_* = 8.61, SD = 1.1; *M_*speech post*_* = 5.75, SD = 1.1) showed a significant difference in SPA compared to the control group (M*_*speech pre*_* = 8.65, SD = 1.1; *M_*speech post*_* = 8.63, SD = 1) in the post-test (*F*(1,34) = 73.07, *p* < 0.001, *η*^2^_*G*_ = 0.68) with the levels during speech after undergoing the training.

**TABLE 1:** Mixed analyses of variance results for self-perceived anxiety and heart rate in the different phases of the Corp-Oral program.

Phase	Measure	Effect	df	MSE	*F*	*p*-value	*η* ^2^_*G*_	Power
Base-line	SPA	Group	1, 17	0.76	1.13	0.302	0.011	0.093
Base-line	SPA	Test	1, 17	1.3	0.09	0.774	0.001	0.093
Base-line	SPA	Group: test	1, 17	1.44	0.43	0.522	0.008	0.081
Anticipation	SPA	Group	1, 17	0.89	43.37	**<0.001**	0.318	0.907
Anticipation	SPA	Test	1, 17	1.1	28.5	**<0.001**	0.273	0.861
Anticipation	SPA	Group: test	1, 17	1.06	32.81	**<0.001**	0.295	0.886
Anticipation	SPA	Control: experimental pre-test	1,34	1.21	0.04	0.837	0.001	0.05
Anticipation	SPA	Control: experimental post-test	1,34	1.23	59.63	**<0.001**	0.637	0.996
Speech	SPA	Group	1, 17	0.61	62.88	**<0.001**	0.404	0.959
Speech	SPA	Test	1, 17	0.98	37.23	**<0.001**	0.393	0.954
Speech	SPA	Group: test	1, 17	0.58	63.47	**<0.001**	0.397	0.956
Speech	SPA	Control: experimental pre-test	1,34	0.63	0.01	0.933	0.001	0.05
Speech	SPA	Control: experimental post-test	1,34	1.027	73.074	**<0.001**	0.682	0.997
Base-line	HR	Group	1, 34	1.14	3031.45	**<0.001**	0.665	0.997
Base-line	HR	Test	1, 34	0.61	9078.21	**<0.001**	0.759	0.999
Base-line	HR	Group: test	1, 34	0.61	250.85	**<0.001**	0.080	0.378
Base-line	HR	Control: experimental pre-test	1	2.23	1138	**<0.001**	0.76	0.999
Base-line	HR	Control: experimental post-test	1	2.789	388.3	**<0.001**	0.52	0.987
Anticipation	HR	Group	1, 34	8.64	0.0	**<0.001**	<0.001	0.05
Anticipation	HR	Test	1, 34	10.34	33.37	**<0.001**	0.348	0.930
Anticipation	HR	Group: test	1, 34	10.34	0.23	0.634	0.004	0.065
Anticipation	HR	Control: experimental pre-test	1	10.3	1.063	0.303	0.002	–
Anticipation	HR	Control: experimental post-test	1	8.798	1.476	0.225	0.004	–
Speech	HR	Group	1, 34	0.39	69.92	**<0.001**	0.444	0.972
Speech	HR	Test	1, 34	0.61	90.29	**<0.001**	0.619	0.995
Speech	HR	Group: test	1, 34	0.61	139.07	**<0.001**	0.715	0.998
Speech	HR	Control: experimental pre-test	1	15.62	88.86	0.017	0.015	–
Speech	HR	Control: experimental post-test	1	11	95.62	**<0.001**	0.21	–

Df, degrees of freedom; MSE, mean squared error; F, F-statistic; *η*^2^_G_, generalized eta squared; SPA, self-perceived anxiety; HR, heart rate. Table showing the results of a mixed ANOVA comparing the effects of group, test, and their interaction (group: test) on self-perceived anxiety and heart rate measures in the base-line, anticipation, and speech phases, as well as on heart rate in the preparation phase. Significant values are highlighted in bold.

**FIGURE 2:**
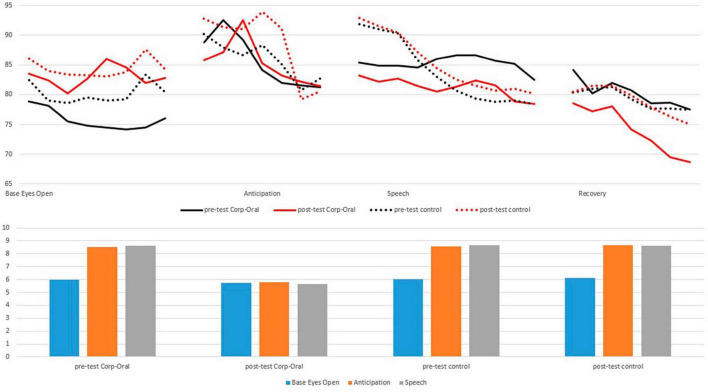
Comparison of heart rate (line graph) and self-perceived anxiety (bar graph) between the different phases of the pre-test and the post-test. In general, both groups showed an increase in heart rate in all post-test phases except the experimental group in the “Speech” post-test phase which showed a decrease in hear rate.

In the case of HR, we present some noteworthy findings from the mixed ANOVA (Table 1). During the “Base-line” phase, a significant interaction between Group and Test was observed (*F*(1,34) = 250.85, *p* < 0.001, *η^2^_*G*_* = 0.080). In the *post hoc* analysis, the experimental group (*M_*basline pre*_* = 76.64, SD = 2.2; *M_*baseline post*_* = 84.14, SD = 2.7) showed a significant difference in HR compared to the control group (*M_*basline pre*_* = 81.45, SD = 3.0; *M_*baseline post*_* = 85.37, SD = 2.4) in both the pre-test (*F*(1,34) = 1138, *p* < 0.001, *η^2^_*G*_* = 0.76) and post-test (*F*(1,34) = 388.3, *p* < 0.001, *η^2^_*G*_* = 0.52). During the “Anticipation” phase, significant differences between the tests were found, *F*(1,34) = 33.37, *p* < 0.001, *η^2^_*G*_* = 0.348. However, no significant interaction between Group and Test was detected (*F*(1,34) = 0.23, *p* = 0.634, *η^2^* = 0.004). In the “Speech” phase, the interaction between Group and Test was highly significant (*F*(1,34) = 139.07, *p* < 0.001, *η^2^_*G*_* = 0.715), indicating a large effect size. Furthermore, *post hoc* analyses showed a significant difference (*F*(1,34) = 95.62, *p* < 0.001) in the post-test, with a large effect size (*η^2^_*G*_* = 0.21) (Figure 2) between the experimental group (*M_*speech pre*_* = 85.21, SD = 1.1; *M_*speech post*_* = 81.24, SD = 1.5) and the control group (*M_*speech pre*_* = 83.81, SD = 5.2; *M_*speech post*_* = 85.21, SD = 4.6).

**FIGURE 3:**
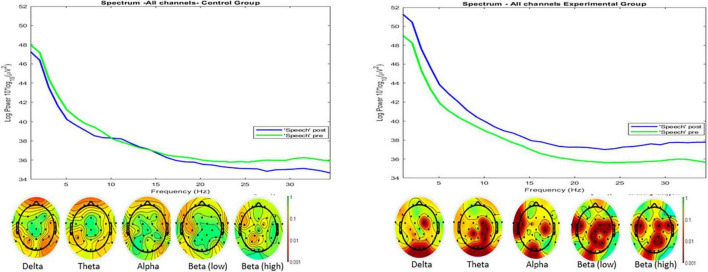
Frequency spectra of all electrodes, for both the control and experimental groups during the Speech phase in the pre-test and post-test, with topographical maps of the scalp areas showing the most significant differences between the pre-test and post-test (according to the *p*-value) for the delta (0.5–4 Hz), theta (4–8 Hz), alpha (8–13 Hz), low beta (13–20 Hz), and high beta (20–30 Hz) frequency bands.

Regarding the EEG results, a significant increase (*Z* = 3.5, *p* < 0.001, *r* = 0.5) was observed in the frequency spectrum of the participants in the Corp-Oral program in the post-test record compared to the pre-test in the “Speech” phase. In the control group, the evidence against the null hypothesis is somewhat less strong (*Z* = −2.98, *p* = 0.03, *r* = −0.1). The positions where this difference was most evident in the experimental group corresponded to delta, theta and beta frequencies in F4, FC2, FC6, FC5, FT9, C4, P4, and Pz. In areas associated with different aspects of language, such as generating sentences or monitoring speech (e.g., middle temporal gyrus or superior temporal gyrus over which sensors such as T7 or T8 are located), no significant changes appear between the pre-test and post-test in either of the two groups. Figure 3 shows the frequency spectra for both the control and experimental groups during the “Speech” phase in the pre-test and post-test. In the lower section of the graphs, topographical maps of the scalp areas showing the most significant differences between the pre-test and post-test can be seen (according to the *p*-value), for the delta, theta, alpha, low beta, and high beta frequency bands.

For the frontal theta/beta ratios, the mixed variance analysis during the “Speech” phase revealed no significant differences. The interaction between the Group and the Test showed a trend, but it was not statistically significant (*F*(1,34) = 2.97, *p* = 0.094, *η*^2^_*G*_ = 0.018). The *post hoc* analysis between experimental (*M_*speech pre*_* = 0.856, SD = 0.59, *M*_*speech post*_ = 0.595, SD = 0.31) and control (*M*_*speech pre*_ = 0.83, SD = 0.48, *M*_*speech post*_ = 0.829, SD = 0.43) groups (*F*(1,34) = 0.57, *p* = 0.457, *η*^2^_*G*_ = 0.013) did not show statistical significance (*p* > 0.05). Although a trend toward a difference in the ratio between the pre-test and the post-test was observed, it did not reach statistical significance (*F*(1,34) = 3.02, *p* = 0.091, *η*^2^_*G*_ = 0.018).

Finally, regarding the frontal alpha asymmetries (FAA), the ANOVA analysis revealed a significant difference between the experimental group (*M*_*speech pre*_ = −0.039, SD = 0.21, *M*_*speech post*_ = 0.358, SD = 0.06) and the control group (*M_*speech pre*_* = −0.043, SD = 0.16, *M_*speech post*_* = −0.027, SD = 0.13) during the “Speech” phase (*F*(1,34) = 20.65, *p* < 0.001, *η*^2^_*G*_ = 0.288), suggesting that both groups displayed different tendencies in terms of approach or avoidance. Moreover, a significant effect between the pre-test and post-test was observed (*F*(1,34) = 46.57, *p* < 0.001, *η*^2^_*G*_ = 0.314), indicating a notable change in the frontal alpha asymmetries of participants between the two tests. The significant interaction between the group and the measure (*F*(1,34) = 39.68, *p* < 0.001, *η*^2^_*G*_ = 0.28) suggests that this change was not uniform across the groups: the experimental group (*M_*F*3*alpha pre*_* = 9.05, *M_*F*4*alpha pre*_* = 8.7; *M_*F*3*alpha post*_* = 9.1, *M_*F*4*alpha post*_* = 13.02), which underwent the anxiety redirection program, displayed a distinct variation in their FAA compared to the control group (*M_*F*3*alpha pr*_*_e_ = 9.11, *M_*F*4*alpha pre*_* = 8.72; *M_*F*3*alpha post*_* = 9.01, *M_*F*4*alpha post*_* = 8.77) when facing the public speaking challenge, with an increase in their FAA.

## 4 Discussion

This brief research report aims to explore some results of evaluating a program for redirecting public speaking anxiety among university students (Corp-Oral).

Similar to other anxiety treatments, for the experimental group, both physiological responses and self-perceived measures were recorded as decreasing (Zeidan et al., 2010; McNally et al., 2013). However, the change in self-perceived measures (33.217% decrease, compared to the 0.231% decrease in the control group) was much more pronounced than in heart rate (4.659% decrease, compared to the 1.67% increase of the control group) during “Speech” phase. We interpret this as a result of the program focusing not so much on decreasing anxiety but on redirecting the activation that it produces, using it to generate a state of greater communicative emphasis and involvement in the discourse.

In both groups, a significant increase in heart rate was observed during the baseline measurement during the post-test. This may be due to an anticipation response (Grupe and Nitschke, 2013), as the participants told us, since they had already gone through the situation in the pre-test. Likewise, in the post-test, the heart rate increased during the “Anticipation” phase, compared to the pre-test. It should be remembered that the control group was reminded of the breathing exercises explained in a previous session in the post-test anticipation phase. In contrast, the Corp-Oral program group was encouraged to engage in activation and redirection techniques involving changes in both bodily posture and emotional state (in line with the program’s principles).

The results could indicate differential physiological and emotional responses between the two groups during the “Speech” phase. How are the experiences between the experimental and control groups reflected at the cortical level?

Given the constraints of the EEG device employed and the experimental conditions, we will interpret the results with caution, emphasizing changes across different frequency bands (it’s important to recognize the limitations of this interpretation, which serves as a foundation for guiding potential hypotheses for future research). As illustrated in Figure 3, the experimental group exhibited the most significant changes, with an increase in spectral power across all frequency bands, predominantly at right fronto-central and parietal electrodes. Following the research of Lapomarda et al. (2022), the increase in delta and theta frequency bands may indicate a reduction in emotional engagement. According to Kirk and Mackay (2003) or Knyazev (2007), theta oscillations might reflect the activation of higher-order cognitive processes related to emotions; more specifically, theta appears sensitive to positive emotion regulation (Zouaoui et al., 2023). Aftanas et al. (2003) found that high-anxiety individuals responded with theta synchronization in posterior regions of the right hemisphere to both threatening and pleasant stimuli. Winter et al. (2020) demonstrated that heightened awareness situations manifest lower heart rates and heightened theta synchronization, along with an increased functional connectivity of the dorsal attention network. Similarly, Aftanas and Golocheikine (2001) evidenced an association between theta and alpha activity and states of internalized attention and positive emotional experience. This aligns with the review by Lomas et al. (2015) which suggests that increased theta and alpha might indicate a state of “relaxed alertness” and inward-directed attention or “intention”. As for beta oscillations, they appear during processes involving a strong endogenous top-down component (Engel and Fries, 2010), as well as during arousal situations (Galvão et al., 2021). However, high beta has been linked to anxiety states (Spitzer and Haegens, 2017; Díaz et al., 2019), more specifically, in right temporal and occipital regions (Pavlenko et al., 2009).

As highlighted, for the experimental group, the sites of spectral power enhancements predominantly point to fronto-central and parietal regions, associated with action planning, sequential moves, error monitoring, response inhibition, self-reflection, and smiling (Dagher et al., 1999; Iwase et al., 2002; Picton et al., 2007; Spaniol et al., 2009; Yalachkov et al., 2009; Owens et al., 2010); and executive control, action sequences, or self-relevance (Crozier et al., 1999; Kübler et al., 2006; Grèzes et al., 2013). Sensorimotor integration, and embodiment (Zotev et al., 2014; Vecchiato et al., 2015; Zanos, 2017; Villafaina et al., 2019). The surge in post-test theta and beta activation at the Pz position is also noteworthy. Arrighi et al. (2016) indicate that given Pz overlies parietal medial cortical areas, oscillations around Pz can tentatively be attributed to the precuneus, an area associated with the integration of internal and environmental information, consciousness processes (e.g., Winter et al., 2020), guided behavior, body schema, and conscious error control (Arrighi et al., 2016). Concurrently, the rise in theta and beta frequencies might hint at improved cognitive processing, attentional commitment, activation, sensorimotor integration, and embodiment (Zotev et al., 2014; Vecchiato et al., 2015; Zanos, 2017; Villafaina et al., 2019) during the public speaking task after participation in the Corp-Oral program. Given the decline in self-perceived anxiety and HR, coupled with the upswing in spectral power across all frequency bands (mainly in the right hemisphere’s fronto-central and parietal electrodes) in the experimental group, the aforementioned studies lead us to infer an enhanced activation and arousal, amplifying top-down control. It is possible that an anxiety state may still be present, despite certain markers such as the increase in high beta. However, due to the reduction in SPA and HR, this increase could also be interpreted as an improvement in the activation or arousal state (Abhang et al., 2016; Galvão et al., 2021). These interpretations are complemented by the results of the theta/beta and FAA ratios.

The results of the frontal theta/beta ratio during the “Speech” phase suggest that, although there was a marginal change in the frontal theta/beta ratio for the experimental group after the program, no conclusive evidence was found for a potential effect of a change in top-down control, consistent with the proposals of van Son et al. (2019). The trend toward a slight decrease in the theta/beta ratio in the experimental group during the post-test might suggest enhanced cognitive control and concentration (Poole et al., 2021). Given the activation triggered by anxiety states (e.g., Sänger et al., 2014), greater voluntary control is expected to address a social stressor, reflected in a decrease in the theta/beta ratio (Wen and Aris, 2020; Poole et al., 2021). In the studied instance, since only the experimental group showed a change in the theta/beta ratio, and given that the participants’ SPA and HR indicate a decrease, it could be inferred that the activation and cognitive control may be attributed to the attitudes of awareness, intentionality, and energy trained in the program.

The frontal alpha asymmetry (FAA) ratio is grounded in two concepts: hemispheric-differentiated emotional processing, with the left frontal region focused on processing positive or approach-related stimuli, while the right processes negative or withdrawal-related stimuli (Davidson, 1992); and the inhibitory influence of alpha on cortical activity, such that lower frontal asymmetry scores (right minus left alpha) are purportedly indicative of less left than right cortical activity (Reznik and Allen, 2018). In terms of the FAA results, considering the literature that associates FAA with approach tendencies or positive motivations and avoidance or negative motivations (e.g., Thibodeau et al., 2006), the findings could suggest that the anxiety redirection program had an impact on how the experimental group participants emotionally processed the public speaking task. This might imply a reduced aversion to the situation of speaking before a panel, given the increased FAA values (Coan and Allen, 2003). This FAA increase is primarily justified by an increase in the right frontal alpha frequency. This suggests reduced right frontal activation, meaning less avoidance (Coan and Allen, 2004). These results would complement previous work (Hofmann et al., 2005; Wang et al., 2015), despite variations in experimental conditions and purposes. In the case of the study by Hofmann et al. (2005), based on previous research on left activation as an indicator of anxiety (Heller et al., 1997), induced distress states and social anxiety traits are linked to an increase in heart rate and greater right hemisphere activity (decrease in FAA) in public speaking situations. Similarly, the study by Wang et al. (2015) shows a decrease in FAA in response to anxiety induced by public speaking. Based on the findings of these studies, it can be inferred that the increase in right frontal activity (rise in FAA) in the post-test for the experimental group, compared to the control group, might be related to control over anxiety during speech.

While we must consider the limitations of these results and the need for future analysis to validate and generalize this study, these findings would suggest that the Corp-Oral program produces small changes in the autonomic nervous system’s reactions to anxiety, but significant differences in attitude and self-perception of state, which at the cortical level are shown in indicators of awareness, cognitive control, activation and intentionality. While these partial results require further exploration and analysis of the program’s long-term effectiveness, they open up an interesting possibility for addressing public speaking anxiety in university students, not focused on reducing it, but on reorienting it using embodied strategies. These strategies could lead to greater action awareness that would cushion the physiological effect of the anxiety response and help generate a better self-perception of the anxiety state.

## Data availability statement

The datasets used and analyzed during the current study are available from the corresponding author on reasonable request.

## Ethics statement

The studies involving humans were approved by the Ethical Committee for Human Studies at the University of Valladolid (code number: PI21-1971). The studies were conducted in accordance with the local legislation and institutional requirements. The participants provided their written informed consent to participate in this study.

## Author contributions

AG-M: Writing – original draft, Writing – review and editing, Conceptualization, Data curation, Formal analysis, Investigation, Methodology, Project administration, Resources, Software, Supervision, Visualization. ES-V: Data curation, Formal analysis, Investigation, Methodology, Visualization, Writing – original draft, Writing – review and editing. LM-Á: Investigation, Methodology, Project administration, Writing – original draft, Writing – review and editing. NB-C: Investigation, Methodology, Project administration, Writing – original draft, Writing – review and editing. SG-R: Investigation, Methodology, Project administration, Writing – original draft, Writing – review and editing.
