# Supplementary material for: Embodied strategies for public speaking anxiety: evaluation of the Corp-Oral program
Source: Front Hum Neurosci. 2023 Nov 27;17:1268798. doi: 10.3389/fnhum.2023.1268798 (PMC10711069; doi:10.3389/fnhum.2023.1268798)
Supplement: Supplementary file 1 [file Image_1.pdf]

Supplementary Material

Article Title

First Author*, Co-Author, Co-Author

*** Correspondence:** Corresponding Author: email@uni.edu

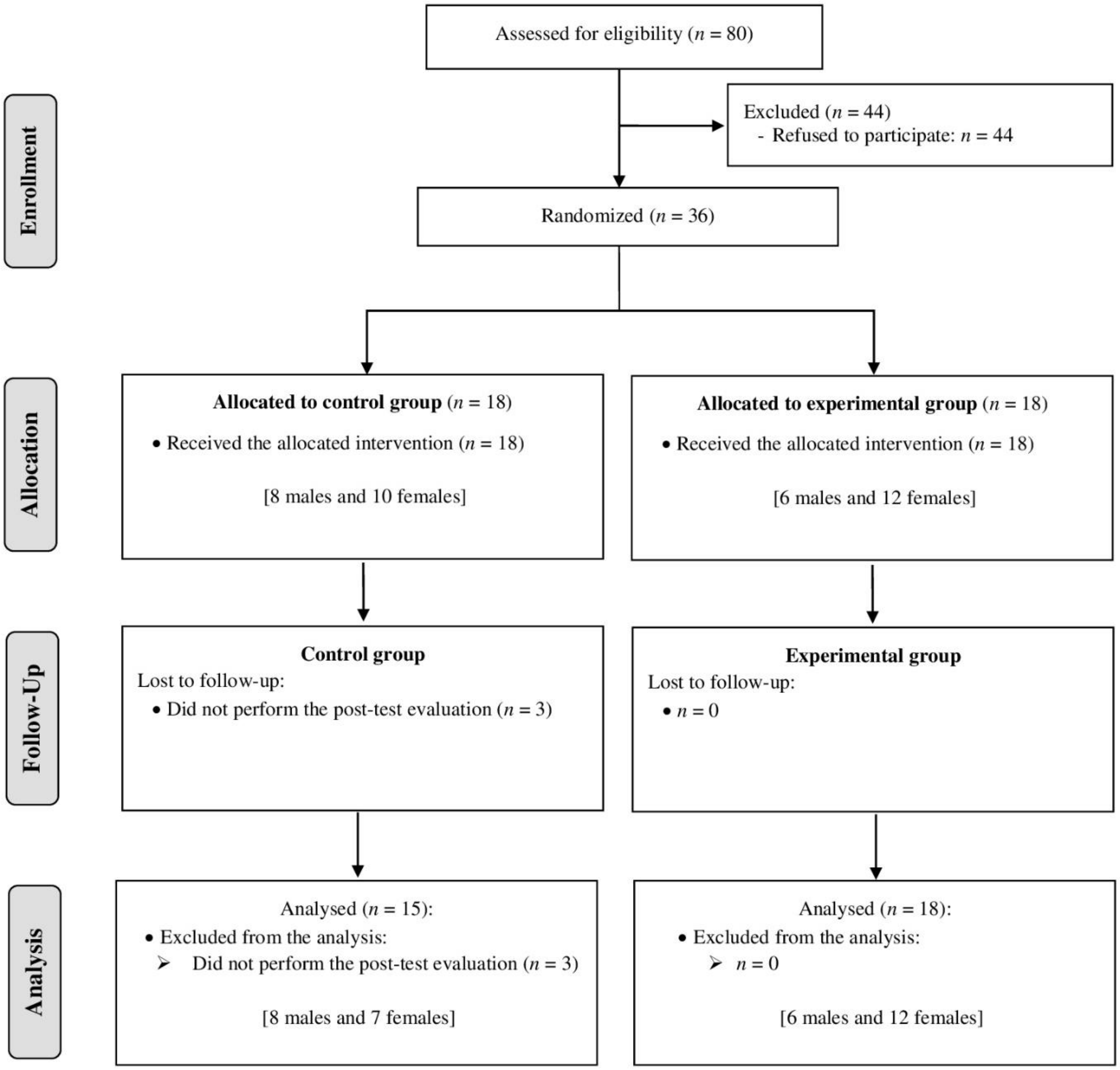

Supplementary Figure 1. Flow chart of participants included in the present study.
